# Case Report: A rare case of sintilimab-induced dermatomyositis in a patient with gastric cancer

**DOI:** 10.3389/fonc.2025.1517391

**Published:** 2025-04-16

**Authors:** Peipei Mou, Fanghua Li, Yingying Wang, Feifei Zhao

**Affiliations:** ^1^ Department of Clinical Laboratory, Shengli Oilfield Central Hospital, Dongying, Shandong, China; ^2^ Department of Oncology, Shengli Oilfield Central Hospital, Dongying, Shandong, China; ^3^ Department of Pharmacy, Shengli Oilfield Central Hospital, Dongying, Shandong, China

**Keywords:** PD-1 inhibitor, immune checkpoint inhibitors, sintilimab, dermatomyositis, immune-related adverse events

## Abstract

The PD-1 inhibitor sintilimab has been approved for the treatment of various malignancies. Here, we reported a rare case of sintilimab-induced dermatomyositis in a patient with gastric cancer and liver metastasis to raise awareness of this serious adverse event. A 64-year-old man presented with the onset of gastric cancer and liver metastasis and received two cycles of sintilimab plus nab-paclitaxel. The patient experienced fever, thrombocytopenia, and rash during the first-cycle treatment, followed by bilateral ptosis, dysphagia, slurred speech, and myalgia during the second-cycle treatment. Elevated muscle enzymes, electromyography, and positive myositis antibodies confirmed the diagnosis of dermatomyositis. He was treated with high-dose corticosteroids and immunoglobulin, resulting in symptom improvement. This case widens the spectrum of immune-related toxicity associated with sintilimab, as well as highlights the need for early recognition and management of these events in patients receiving ICIs.

## 1 Introduction

Immune checkpoint inhibitors (ICIs) have revolutionized the current oncology era, targeting the immune system components to fight tumor cells (1). Currently, multiple ICIs, such as PD-1/PD-L1 inhibitors (nivolumab, pembrolizumab, sintilimab) and CTLA4 inhibitors (ipilimumab, tremelimumab), have been widely used for improving patients survival across a variety of malignancies (2). Despite remarkable therapeutic outcomes, ICIs could induce immune-related adverse events (irAEs) due to excessive immune activation (3). The common events are systemic, gastrointestinal or endocrine toxicities, ranging from mild to severe (4). Most irAEs are self-limiting, but severe irAEs are fatal in some cases (5). However, certain irAEs are less common and remain poorly understood, which pose significant challenges in clinical practice due to their unpredictable onset and lacking established management strategies (6). The rare grade 3 or higher irAEs, such as neurotoxicities, cardiomyotoxicities, and hematologic events, were associated with a tendency for inferior overall survival (7, 8). Among them, ICI-induced dermatomyositis represents a subset of irAE that occurred during ICI therapy and has been documented in cases received PD-1 inhibitors (e.g., nivolumab, pembrolizumab) (9–12), PD-L1 inhibitors (e.g. atezolizumab, avelumab) (13, 14), or CTLA-4 inhibitor ipilimumab (15). Given the emerging recognition of irAEs, providing any information on their clinical characteristics, diagnostic approaches, and management strategies is of significant value, particularly considering their rarity and unpredictability.

Sintilimab is a fully human IgG4 monoclonal antibody against programmed cell death receptor-1 (PD-1), blocking the interaction of PD-1 with its ligands and restoring the endogenous antitumor T-cell response (16). Currently, sintilimab has been approved or under investigation for treating multiple malignancies (16). The clinical trials have well documented the manageable safety profile of sintilimab (17), which commonly includes fever and immune-related hypothyroidism, thyroid stimulating hormone increased, free thyroxine decreased (16). Sintilimab have been reported to induce rare and fatal ICI-related myocarditis (18). However, to date, the information about ICI-related dermatomyositis following sintilimab treatment has not been available, which highlights the critical need for further attention to the full spectrum of irAEs associated with sintilimab and the necessity for increased vigilance in identifying and managing such rare toxicities. Herein, we report the clinical and prognostic characteristics of the first case with dermatomyositis induced by sintilimab to expand its irAE spectrum and provide information on clinical characteristics, diagnosis, and management of this uncommon but potentially serious side effect. By sharing this experience, we aim to raise awareness among clinicians and contribute to better understanding and management of ICI-induced dermatomyositis in cancer patients.

## 2 Case description

**Figure 1:**
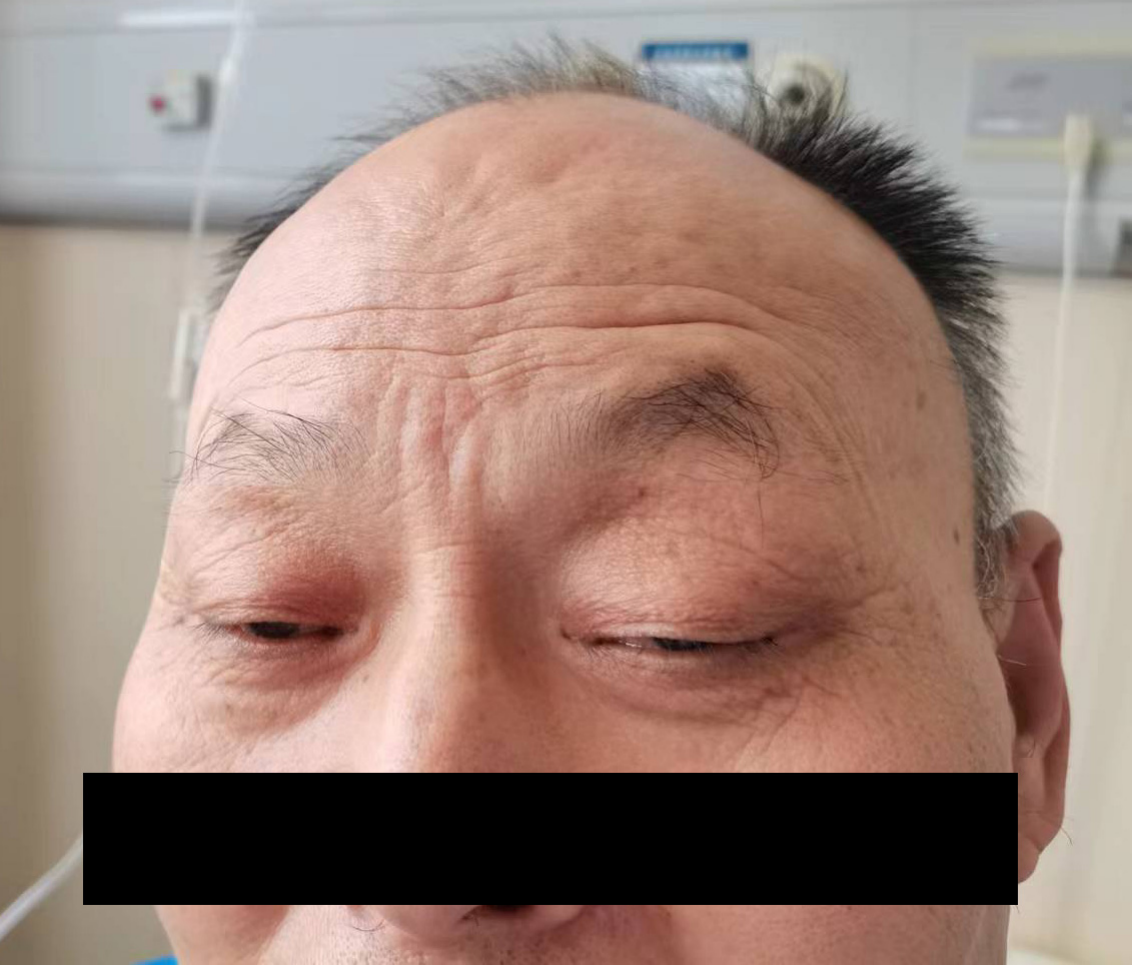
The patient presented with bilateral eyelid ptosis.

**Figure 2:**
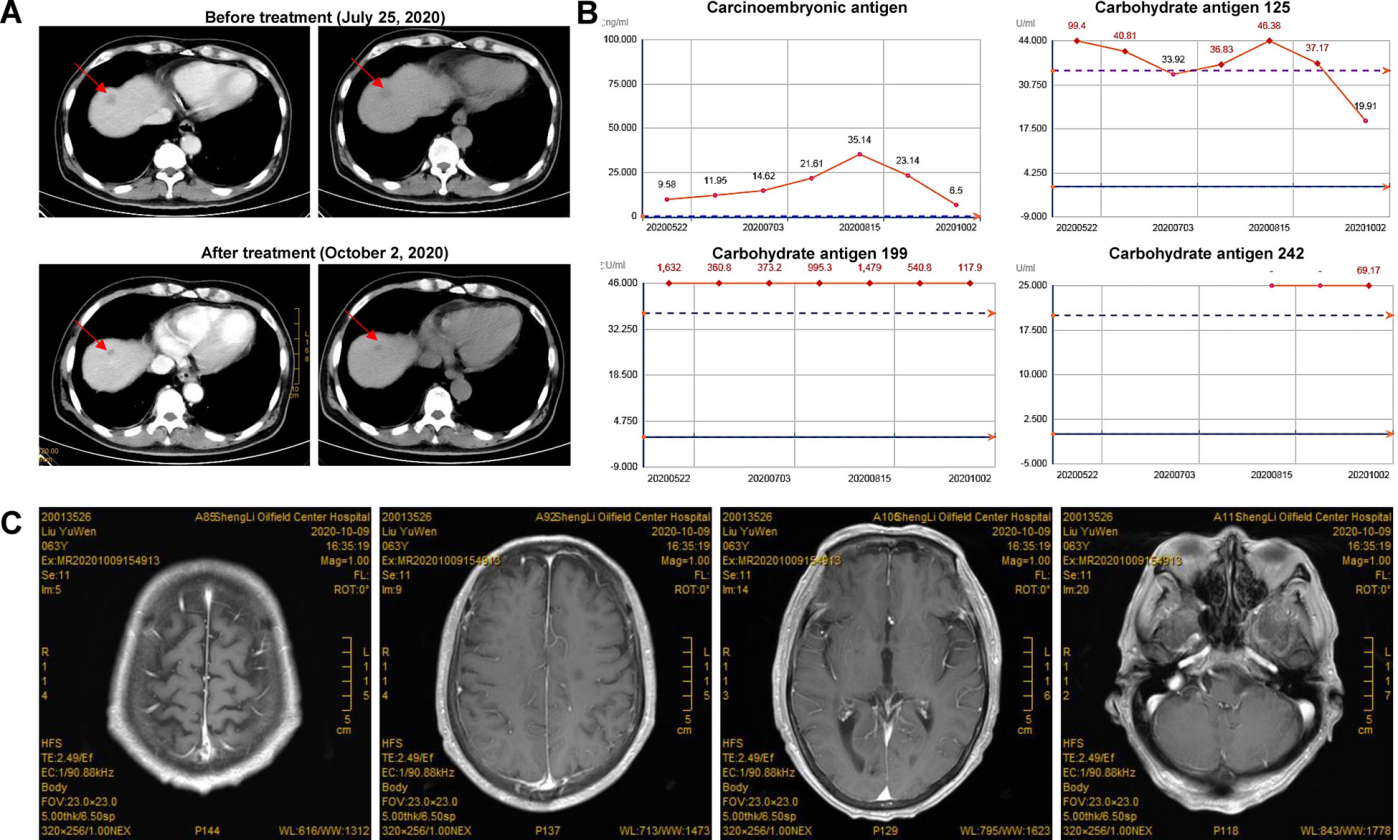
Tumor and biomarkers change in a patient with dermatomyositis after sintilimab plus nab-paclitaxel. **(A)** The abdominal CT indicated a reduction in liver masses after treatment; **(B)** The levels of tumor markers were decreased after treatment. Carcinoembryonic antigen, carbohydrate antigen (CA) 125, CA 199, and CA 242 were detected with electrochemiluminescence assay using commercial kits (Roche Diagnostics GmbH, Penzberg, Germany). **(C)** Enhanced brain MRI revealed the old cerebral infarction and white matter ischemic changes, excluding the central nervous system metastasis.

A 64-year-old male patient was first (April 2, 2020) admitted to our hospital due to upper gastrointestinal hemorrhage and underwent the surgical resection of gastric cancer and liver metastasis. Postoperative pathology confirmed moderately to poorly differentiated adenocarcinoma. After four cycles of adjuvant chemotherapy with oxaliplatin and S-1, the abdominal CT indicated an enlarged liver mass, leading to the second admission; thus, sintilimab plus nab-paclitaxel was administered on August 14, 2020. During the first cycle of sintilimab plus nab-paclitaxel, he developed fever (39°C), thrombocytopenia, and rash, which did not recur during the second cycle. At the third admission (October 1, 2020), the patient presented with bilateral eyelid ptosis, but without obvious abnormal vital signs and rash. On the next day, he developed progressive bilateral ptosis, impaired upper eyelid function (**Figure 1**), dysphagia with drooling, slurred speech, numbness of limbs, and myalgia of lower extremities, aggravated muscle weakness. Despite the neuromuscular symptoms, abdominal contrast-enhanced CT showed a reduction in liver lesion size (**Figure 2A**), and the electrochemiluminescence assay showed decreased levels of tumor markers (including carcinoembryonic antigen, carbohydrate antigen [CA] 125, CA 199, and CA 242; **Figure 2B**) (19), precluding the suspicion of paraneoplastic syndrome due to tumor progression. Enhanced brain MRI revealed the old cerebral infarction and white matter ischemic changes, excluding the central nervous system metastasis (**Figure 2C**).

Serum biochemical tests (**Table 1**) showed elevated alanine aminotransferase (ALT), aspartate aminotransferase (AST), creatine kinase (CK), and lactic dehydrogenase (LDH), raising suspicion for irAEs (20). Thus, the patient received supportive care and low-dose prednisone (0.5 mg/kg/day) on day 2 of hospitalization. On October 14, 2020, myocardial enzyme examination showed CK of 807 U/L, CK-MB of 69 U/L, LDH of 541 U/L, and AST of 96 U/L (**Table 1**). Echocardiography suggested mild aortic regurgitation and reduced left ventricular diastolic function (ejection fraction 67%). Acetylcholine receptor antibody testing with enzyme-linked immunosorbent assay was negative, excluding the possibility of myasthenia gravis. Immunoblot testing of myositis antibodies showed a weak positive for PM-Scl75 and a positive for signal recognition particle (SRP) antibodies, which is more indicative of ICI-related myositis or dermatomyositis rather than classical polymyositis. Electromyography demonstrated occasional short-range, small-amplitude, polyphasic motor units, suggesting myopathic damage and further supporting an immune-mediated inflammatory myopathy. Based on clinical symptoms and laboratory findings, a diagnosis of dermatomyositis as a sintilimab-induced irAE was established. In accordance with the 2019 CSCO guidelines for managing ICIs-related toxicities, methylprednisolone (1-2 mg/kg/day) and immunoglobulin (400 mg/day) were administered intravenously for 5 days. The patient’s clinical symptoms were significantly improved, with improved bilateral eyelid elevation, alleviated dysphagia, reduced drooling, clear speech, and reduced lower limb pain. After significant symptomatic and clinical improvement, treatment was changed to oral methylprednisolone and tapered gradually. The patient was discharged on October 24, 2020, and no recurrence of dermatomyositis symptoms was noted at the last follow-up (November 25, 2020).

**Table 1:** Laboratory results in this case with dermatomyositis.

Laboratory parameters	Admission	Hospital day 14	References
Alanine aminotransferase (U/L)	62 ↑	43 ↑	7-40
Aspartate aminotransferase (U/L)	123 ↑	96 ↑	8–40
Creatine kinase (U/L)	2134 ↑	807 ↑	50–170
CK-MB (U/L)	81 ↑	69 ↑	0–24
Lactic dehydrogenase (U/L)	480 ↑	541 ↑	120–250

↑indicated the increase from reference values.

## 3 Discussion

Dermatomyositis is a rare inflammatory myopathy with cutaneous manifestations and severe systemic symptoms that can occur spontaneously, as paraneoplastic syndrome or as a drug reaction (e.g., ICIs) (21). Several cases with dermatomyositis have been reported sporadically as a rare irAE related to other ICIs (22). This is the first known case of sintilimab-induced dermatomyositis. The patient with gastric cancer developed dermatomyositis after sintilimab treatment and improved with corticosteroids and immunoglobulin treatment. Our report expanded the spectrum of irAE caused by sintilimab and raised clinicians’ awareness about this event.

According to previous reports, the clinical presentations of dermatomyositis are highly variable (**Table 2**), commonly including skin manifestations (e.g., rash) and muscle weakness (13, 23). In this case, the patient presented with classic symptoms of dermatomyositis, including progressive bilateral eyelid ptosis, dysphagia, muscle weakness, and limb pain, but without significant systemic manifestations such as fever and rash. Notably, the absence of cutaneous signs initially made diagnosis challenging. Therefore, further laboratory findings, including elevated myocardial enzymes (CK, AST, LDH) and positive myositis-specific antibodies (PM-Scl75, SRP), supported the diagnosis of dermatomyositis. Electromyography further confirmed muscle damage. Although clinical, biochemical, and electrophysiological findings strongly support the diagnosis of dermatomyositis, the lack of muscle biopsy still limits the ability to provide histopathological confirmation of the diagnosis.

**Table 2:** Representative cases with dermatomyositis induced by different checkpoint inhibitors.

Authors, years	ICIs	Sex/age	Tumor	Therapy cycles	Clinical findings	EMG	Myositis specific aAbs	Laboratory findings	Treatment	Outcome
Our case	Sintilimab	M, 64	Gastric cancer	1	Bilateral eyelid ptosis, dysphagia with drooling, slurred speech, numbness of limbs, myalgia of lower extremities, muscle weakness	Myopathic pattern	PM-Scl75 positive; Anti-SRP positive.	Elevated CK (2134 U/L), AST (123 U/L), lactic dehydrogenase (480 U/L)	Methylprednisolone + immunoglobulin	Improvement in symptoms
Berger et al., 2018 (9)	Pembrolizumab	M, 83	Metastatic melanoma	6	asthenia, weakness, swallowing disorder, rash	Myopathic pattern	Anti TIF-1γ positive	Elevated CK (1883 U/L), aldolase (15.5 UI/L, AST (221 UI/L)	High-dose corticotherapy + immunoglobulin	Improvement in symptoms
Wiggins et al., 2021 (10)	Pembrolizumab	M, 74	Squamous cell lung carcinoma	NA	Rash, muscle weakness, periorbital edema, fatigue, chills, wheezing	NA	NA	Elevated CK (247 U/L)	Prednisone + immunoglobulin	Improvement in symptoms
Kudo et al., 2018 (11)	Nivolumab	M, 42	Lung adenocarcinoma	3	fatigue, muscle weakness, rash, shawl sign, periungual erythema	Myopathic pattern	Anti-Jo-1 and anti-ARS antibodies negative	Normal CK (137 U/L); Elevated aldolase (23.7 U/L)	Prednisolone	Symptoms were slightly and temporarily improved.
Asano et al., 2021 (12)	Nivolumab	M, 15	Nasopharyngeal cancer	7	Facial erythema, muscle weakness, myalgia, rash, Gottron’s sign, periungual erythema, nailfold bleeding	NA	Anti TIF-1γ positive	Elevated CK (2600 U/L) and aldolase (27.1 U/L)	prednisolone + methotrexate + gamma globulin	Improvement in symptoms
Yamaguchi et al., 2021 (14)	Atezolizumab	M, 75	Small-cell lung carcinoma	1	Rash, myalgia, motor weakness, Gottron’s sign	Myopathic pattern	Anti-Jo-1 and anti-SRP antibodies negative; Anti TIF-1γ positive	Elevated CK (5698 U/L), myoglobin (1257 μg/mL), and aldolase (51.5 U/L)	Prednisolone	Improvement in symptoms
Lin et al., 2020 (24)	Toripalimab	M, 66	Melanoma	2	Muscle pain and weakness, dysphagia, rash, purplish-red scaly maculopapules, poikiloderma, occasional pruritus	Myopathic pattern	NA	Elevated CK (654 U/L), lactic dehydrogenase (259 U/L).	prednisone + methylprednisolone	Symptoms were controlled
Li et al., 2023 (32)	Camrelizumab	M, 59	oesophageal cancer	7	Swollen face, erythema papules, pruritus, weakness	NA	Anti TIF-1γ positive	Elevated fibrinogen, erythrocyte sedimentation rate, C-reactive protein.	methylprednisolone + immunoglobulin + cyclophosphamide	Symptoms were relieved.

EMG, electromyography; aAbs, autoantibodies; M, male; F, female; NA, not available; Anti-TIF-1γ, anti-transcription intermediary factor 1-gamma; CK, creatinine kinase; AST, aspartate minotransferas.

Because of its complex pathogenesis, dermatomyositis was often mistaken as a paraneoplastic syndrome (24). Here, the absence of tumor progression on imaging confirmed its causality with sintilimab. Despite the diagnosis of sintilimab-induced dermatomyositis, which may raise concerns about the impact on antitumor efficacy, our findings indicate that sintilimab’s therapeutic effects were not compromised by the development of dermatomyositis in this case, as evidenced by tumor shrinkage and decreased tumor markers. This suggests that irAEs may not necessarily interfere with the therapeutic benefit of ICIs, as also observed in a previous study (25). Overall, the complexity of clinical presentations highlights the importance of comprehensive diagnostic evaluation, including clinical, biochemical, and electrophysiological assessments, in patients receiving ICIs, especially those without cutaneous signs.

Although the exact mechanism of ICIs-induced dermatomyositis remains unclear, previous studies have supposed that irAEs were drived by several possible mechanisms (26). The excessive T-cell activation and loss of immune tolerance triggered by ICIs may lead to the activation of other immune regulators, causing eventual muscle inflammation (27). Additionally, the direct molecular mimicry and the emergence of myositis-specific autoantibodies (26), such as anti-SRP and anti-PM-Scl (which were detected in this patient), may play a role in triggering immune-mediated muscle damage. However, these mechanisms are speculative, and further research is needed to fully understand the underlying pathophysiology of ICI-induced dermatomyositis.

The management of dermatomyositis typically involves immunosuppressive therapy, using systemic corticosteroids as a mainstay of therapy (28). Similarly, our case responded well to corticosteroid treatment, consistent with guidelines for managing irAEs (29). Additionally, intravenous immunoglobulin was administered, which has been shown in the literature to enhance recovery in cases of dermatomyositis by improving muscle function and reducing inflammation (30). This combined immunosuppressive approach led to significant symptom relief and functional recovery in the patient, emphasizing the importance of early and aggressive intervention in managing severe irAEs. However, close monitoring for potential steroid-related side effects and long-term follow-up are necessary to ensure sustained remission.

Although clinical guidelines for irAE diagnosis and management exist, specific recommendations for ICI-related dermatomyositis remain limited due to its rarity. In a review of 22 cases by Guerra NL et al. (13), this event usually develops early in the course of treatment (mostly within 1-4 cycles). Thus, when a patient receiving ICIs presents with skin manifestations, muscle weakness, or myalgias, it is recommended early and thorough examinations, such as physical status, skin examination and biopsy, electromyography and muscle biopsy; if needed, imaging assessment, a biological checkup (e.g., creatinine kinase, aldolase) and myositis-specific autoantibodies, which will help to eliminate the possibility of paraneoplastic syndrome or other autoimmune diseases. Based on existing literature and clinical experience (20, 31), we preliminary summarized the severity classification and treatment strategies for ICI-induced dermatomyositis (**Table 3**). Although serious dermatomyositis is rare, in such severe cases, ICIs should be permanently discontinued; the patient should be hospitalized immediately and receive symptomatic treatment under the supervision of a specialized physician.

**Table 3:** Management and recommendations for ICI-induced dermatomyositis.

Severity	Clinical Manifestations	Examinations	Recommended Treatment (grade I)	Recommended Treatment (grade II)
Grade 1	Mild muscle weakness, with or without pain.	Thorough physical and laboratory examinations.	• Continue ICIs; • Consider prednisone at an initial dose of 0.5 mg/kg for patients with elevated CK and/or aldolase levels accompanied by muscle weakness; • In patients with pain, acetaminophen or NSAIDs should be started for pain management after excluding contraindications. • Interrupt statins.	
Grade 2	Moderate muscle weakness, with or without pain; limiting instrumental ADL.	Detailed medical history, physical examination; hematological tests; electromyography. MRI is recommended for cases of suspected joint involvement.	• Interrupt ICIs until recovery from symptom, CK returned to normal level, and prednisone dose reduced to <10 mg; if symptom aggravated, analgesia with NSAIDs is recommended according to the management of grade 3 events after excluding contraindications. • If CK ≥ 3 ×UNL, initiate steroids (e.g. 0.5-1 mg/kg prednisone or equivalent) • Consultation with a rheumatologist or neurologist.	• Consider permanently discontinue ICIs for symptomatic patients or physical/biological abnormalities (CK increased, or abnormalities in electromyography, MRI or biopsy). Consider restart ICIs when CK returned to normal level or clinical symptoms improved.
Grade 3-4	Severe muscle weakness, with or without pain; limiting self-care ADL.	Detailed medical history, physical examination; hematological tests; electromyography. MRI is recommended for cases of suspected joint involvement.	• Interrupt ICIs until the AE has reverted to grade 1; In case of impaired myocardium, permanently discontinue ICI. • In the case of severe symptoms, admit the patient. • Consultation with a rheumatologist or neurologist. • immediately start methylprednisone (1 mg/kg) or equivalent • If severe symptoms (e.g., profound weakness leading to activity limitation, or cardiac, respiratory, or dysphagia involvements), start high-dose methylprednisolone (1–2 mg/kg) intravenously or pulse therapy.	• Consider intravenous immunoglobulin. • For severe or acute cases, plasmapheresis should be considered after consultation with rheumatologists or neurologists.

ICI, immune checkpoint inhibitor; ADL, activity of daily living; CK, creatinine kinase; NSAIDs, nonsteroidal anti-inflammatory drugs; UNL, upper normal limit; MRI, magnetic resonance imaging; AE, adverse event.

In summary, this report describes the first documented case of sintilimab-induced dermatomyositis, underscoring the rarity of this irAE. The favorable outcomes of this patient demonstrate the importance of prompt diagnosis and treatment. This report recommends that clinicians should be aware of its early manifestations and therapeutic regimens, given the rarity and complexity of ICI-induced dermatomyositis. Nevertheless, as this report is based on a single case, further investigations are needed to expand understanding of its clinical presentation, optimal management strategies, and long-term prognosis. Given the rarity and complexity of ICI-induced dermatomyositis, future research involving larger datasets and mechanistic studies is warranted to provide insights into its pathophysiology. Additionally, as the use of ICIs continues to expand across different cancer types, optimizing treatments is the priority to minimize morbidity and mortality of this complication.

## Data Availability

The original contributions presented in the study are included in the article/Supplementary Material. Further inquiries can be directed to the corresponding authors.
